# Geriatric Assessment as an Important Tool for Post-Hip Surgery Prognosis in Seniors

**DOI:** 10.3390/nursrep15070262

**Published:** 2025-07-17

**Authors:** Anca Iuliana Pîslaru, Irina Sîrbu, Sabinne-Marie Albișteanu, Ramona Ștefăniu, Ana-Maria Turcu, Gabriela Grigoraș, Iulia-Daniela Lungu, Roxana Maria Pînzaru, Ioana Dana Alexa, Adina Carmen Ilie

**Affiliations:** 1 Department of Medical Specialties II, Faculty of Medicine, University of Medicine and Pharmacy “Grigore T Popa”, 700115 Iasi, Romaniaana-maria_turcu@umfiasi.ro (A.-M.T.); grigoras_gabriela@d.umfiasi.ro (G.G.); lungu.iulia-daniela@d.umfiasi.ro (I.-D.L.);; 2 Department of Orthopaedics and Traumatology, “Sf. Spiridon” Emergency County Hospital, 700111 Iasi, Romania

**Keywords:** senior patients, hip fracture, recovery, geriatric assessment

## Abstract

Hip fractures in elderly patients pose significant clinical challenges, confronting us with high morbidity and mortality rates. A comprehensive geriatric assessment plays an important role in determining prognosis as well as the indication for surgery. **Aim**: In this study, we aim to (1) assess frailty-based functional status in seniors with hip fractures, (2) evaluate geriatric assessment’s predictive value for postoperative recovery, and (3) analyze 1-year postoperative survival. **Material and Methods**: This prospective study included 60 senior patients admitted for hip fracture in the Orthopedics Department. Patients were examined using geriatric assessment instruments Mini Mental State Examination (MMSE), Geriatric Depression Scale (GDS), Mini Nutritional Assessment (MNA), and Frailty Groningen Indicator (GFI). We recorded the sex, marital status, number of comorbidities, and number of recommended drugs. **Results**: In total, 65% of patients were frail pre-surgery; the proportion increased post-surgery to 86.7%; (*p* = 0.005). Age greater than 80 years and unmarried marital status were associated with higher frailty risk (*p* = 0.04; *p* = 0.03). Preoperatively, important predictors of frailty were mild–moderate cognitive impairment (*p* = 0.017), mild–moderate depression (*p* = 0.01), and malnutrition (*p* = 0.04). Postoperatively, only mild–moderate cognitive impairment (*p* = 0.04) and mild–moderate depression (*p* = 0.01) proved to be important predictors of frailty. According to the ROC curve, good predictors of postoperative frailty were shown to be preoperative frailty and the degree of polypharmacy and comorbidity. Of all parameters predictive of postoperative frailty, only the number of medications reached statistical significance (*p* < 0.038). The study identified a 1-year all-cause mortality rate of 42.6% in elderly patients who underwent hip fracture surgery, with a significant association between mortality and preoperative MMSE, GDS, and MNA scores. **Conclusions**: Complex geriatric assessment of senior patients with hip fracture can stratify postoperative risk and predict 1-year mortality and postoperative functional recovery. Key predictors include cognitive status, depression, malnutrition, and comorbidities. Multidisciplinary care and standardized evaluation are essential for improving outcomes.

## 1. Introduction

Hip fractures are the most common traumatic event in the geriatric population, and their rate has increased steadily in recent decades. The worldwide annual incidence of hip fractures is predicted to rise from 1.66 million in 1990 to 6.26 million in 2050 [1]. In the United States alone, there are 250,000 new cases each year [2]. Beyond their clinical impact, hip fractures place a substantial economic burden on healthcare systems worldwide, due to the high cost of acute treatment, long-term rehabilitation, and institutional care. Moreover, these injuries lead to significant loss of independence and increased need for social support and pose a growing public health and socio-economic challenge [3,4].

Older adults are distinctly vulnerable to adverse consequences in surgical treatment and subsequent recovery. This often leads to increased disability, institutionalization, and mortality. Previous research indicates that using a standardized multidisciplinary approach for treating hip fractures in elderly patients enhances care quality while reducing hospital stays, postoperative complications, and mortality rates [3].

Studies have determined the in-hospital mortality rate in patients undergoing surgery for hip fracture to range from 1.52 to 11.4%, while other studies have projected a 25% decrease in life expectancy after hip fracture [4,5,6]. Previous data identified numerous risk factors for hip fracture complications, including age, male sex, low preoperative mobility, fracture type, and time to surgery [4,5,6,7]. Essential factors that can improve functional outcomes in older patients undergoing hip fracture surgery include thorough preoperative evaluations, timely surgery, structured recovery and rehabilitation goals, early mobilization, and medication management. Comorbidities associated with increased risk of post-fracture frailty and negative prognosis include heart failure, atrial fibrillation, diabetes, dementia, and renal failure [8,9,10,11,12,13,14].

Frailty, a hallmark of the aging years, is too often viewed narrowly through the mechanisms of physical degradation. Frailty is not just a state of vulnerability but an attack on the homeostasis of multiple physiological systems, mediated by one or more stressful events, defined, in the last resort, as a cumulative decline recorded throughout life [1,15].

Frailty with its high prognostic value is important in primary care, where it serves as a diagnostic factor in many therapeutic decisions [16]. Frailty assessment should be widely incorporated into future clinical practice for geriatric patients, as early interventions and appropriate care for frail seniors may help prevent poor outcomes [11]. Frailty is as a predictor of adverse outcomes, including fall risk, disability, postoperative complications, institutionalization, and mortality [4,17]. Although there are numerous frailty indices, the Groningen Frailty Indicator (GFI) is recognized worldwide, incorporating physical, psycho-emotional, and social components. A total score of 4 or higher is considered positive for frailty [18].

A comprehensive geriatric evaluation is urgently needed to mitigate disability, address comorbidities, and reduce mortality in frail patients with hip fractures. Orthogeriatric care, which employs a multidisciplinary team including an orthopedic surgeon, geriatrician, geriatric nurse, and physiotherapist, has been studied in the management of proximal femoral fracture.

Polypathology, polypharmacy, and the degree of disability are among the conditions that make the care of elderly patients particularly challenging, requiring a thorough geriatric assessment within a multidisciplinary team, where each member plays a crucial role in the clinical course and prognosis of the patient. Even more, a traumatic event such as a hip fracture, involving both a significant physical burden and the psychological impact associated with the fear of recurrence, calls for complex and coordinated geriatric care.

Such care involves the systematic use of geriatric and clinical assessment tools to clearly identify an older adult’s profile, covering nutritional, psycho-emotional, and cognitive status, the degree of frailty and functional dependence, the number of comorbidities, and polypharmacy. Such individualized profiling is fundamental to establishing a tailored recovery plan.

Within this multidisciplinary framework, the geriatric nurse has crucial responsibilities, including comprehensive pain assessment, the management of acute and chronic comorbidities, and ensuring accurate administration of analgesic medication. They strive to prevent complications associated with immobility syndrome (such as pressure ulcers, aspiration pneumonia, muscular atrophy, and joint stiffness) and evaluate patient safety (e.g., fall and injury risk). Further, the nursing care plan must be adapted to the diagnoses established through both medical and geriatric evaluations. It has shown promising results in these patients, with reduced mortality and readmission rates [3,8,9,10,11,12,13] and fewer postoperative complications [11,19,20]. Cognitive frailty refers to the simultaneous presence of both cognitive impairment and physical frailty. Many studies identify an association between cognitive impairment and physical frailty [21,22,23].

Numerous research studies have shown that physical frailty, psychological distress, and cognitive impairment are inter-related contributors to adverse events. Some data reported that both cognitive impairment and physical frailty are linked to the risk of falling, with hip fracture among the severe consequences for the older adult. Postoperative delirium is the most common type of complication after hip fracture. Delirium during the perioperative period can significantly impact a patient’s treatment effectiveness, increasing mortality risk, the need for home care after discharge, hospital stay duration and the likelihood of hospital-acquired complications [5,6]. Although frailty is associated with poor outcomes for post-hip fracture recovery, studies show that anxiety and depression also exacerbate the decline of older patients with hip fractures [24]. Other data show that frailty is the most important predictor of depression and anxiety in this population, often linked to fear of falling. Some results indicate that depressed older patients are more susceptible to frailty in post-surgical recovery after hip fracture [25,26].

Although some data record an increase in mortality in those for whom surgery is delayed beyond 48 h, frailty assessment has an important role in establishing the indication for surgery, enabling improved survival and even quality of life [27,28,29]. Further research is needed on the efficacy of nonoperative treatment in frail older adults. For example, the current literature is insufficient for adequately counseling patients and their relatives about alternatives to hip fracture surgery [30,31,32,33].

At the same time, most studies show that malnutrition and frailty are important predictors of postoperative complications and poor recovery in older persons who have undergone hip fracture surgery [34,35,36,37,38]. The importance of using the MNA (Mini Nutritional Assessment) in frailty assessments of older persons has been emphasized, demonstrating that nutritional status influences the degree of frailty [39].

Data on the role of geriatric assessment in predicting frailty in hip fracture patients remain limited. This study aims to determine the functional status of senior patients with hip fractures by using pre- and postoperative geriatric assessment. Specifically, the objectives are to (1) assess frailty-based functional status, (2) evaluate the predictive value of geriatric assessment for postoperative recovery, and (3) analyze postoperative 1-year survival. Thus, we want to obtain a clearer understanding of an optimal recovery of locomotor capacity that would ensure the reintegration of patients into their socio-familial environment with a quality of life as close as possible to their expectations.

We hypothesize that frailty, as evaluated by comprehensive geriatric assessment, best reflects functional status, rehabilitation capacity, and survival in elderly patients with hip fractures.

We can consider the senior patient with a hip fracture a paradigm of frailty, which becomes a challenge for both geriatricians and orthopedists. Therefore, a multidisciplinary approach, with the development of common objectives and protocols, is essential in the management of these patients, with the aim of reducing the risk of mortality and frailty both in the short and long term. Early identification of patients at high risk of mortality requires a complex geriatric assessment, addressing all of their characteristics and their resultant frailty.

Thus, the complex geriatric assessment is under-utilized in predicting post-surgical outcomes in hip fracture patients.

## 2. Materials and Methods

### 2.1. Study Design and Setting

This is a prospective cohort study using a descriptive–correlational design. Data were collected from 2019 to 2020, and the study is ongoing. The manuscript is intended to present a planned interim analysis, in accordance with the approved research protocol. Although not registered as a formal clinical trial, this study followed a predefined, ethics panel-approved protocol, including this interim analysis focused on early postoperative outcomes. The study is structured in several phases, and this initial analysis aims to evaluate preliminary correlations between geriatric assessment scores and postoperative outcome parameters, including 1-year mortality (defined as death within 12 months post-surgery), among the first group of enrolled participants. The study is designed to continue into additional phases that include a longer follow-up period and extended analysis of a larger sample. The manuscript is performed in accordance with the Declaration of Helsinki Ethical Principles for Medical Research and approved by the Ethics Committee of Saint Spiridon University Hospital, Iași, with a collaboration agreement with Clinical Hospital Dr. C. I. Parhon, Iași.

The study begins after receiving approval from the Hospital Ethics Committee. Patients received a preoperative examination within 24 h of admission using widely recognized geriatric scales and questionnaires conforming to international standards. Cognitive function was assessed using the Mini Mental State Examination (MMSE) and psycho-emotional status with the Geriatric Depression Scale (GDS). Nutritional status was determined with the Mini Nutritional Assessment (MNA), and frailty was classified using the Groningen Frailty Indicator (GFI). The geriatric assessment was repeated 72 h after surgery by the same geriatrics team.

### 2.2. Study Population

**Figure 1:**
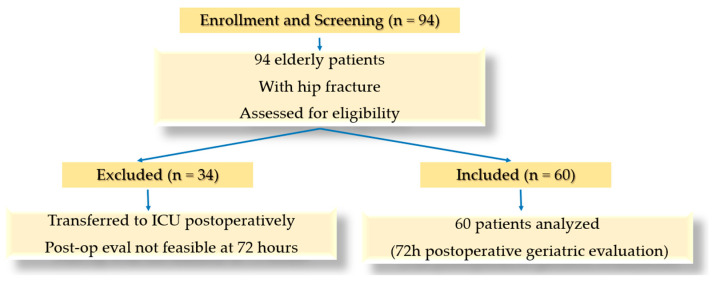
Flowchart for patient inclusion and attrition.

This paper presents only the data collected between 2019 and 2020. It includes an initial group of 94 older patients (>60 years) who had suffered a hip fracture and were scheduled for surgery. Of the 94 patients, we evaluated 60 patients at 72 h postoperatively. This timing may limit the sensitivity of detecting meaningful changes in domains such as nutritional status and cognition, as short-term tools like the MNA are not designed to capture the slower biological and functional changes that occur beyond this early postoperative period. The remaining 34 were not evaluated as their postoperative condition deteriorated, and they were transferred to the Intensive Care Unit due to acute complications frequently observed in frail seniors following hip surgery. These complications included respiratory failure, cardiovascular instability, embolic events, and severe infections leading to sepsis. We acknowledge that this may introduce selection bias, as patients with more severe postoperative complications were not included in the postoperative assessment. This potentially affects the generalizability of the results. Although the baseline characteristics of these patients (e.g., age, comorbidities, and preoperative frailty scores) were comparable to those of the analyzed cohort, the clinical severity of their postoperative complications clearly placed them at higher risk for adverse outcomes (Figure 1).

#### 2.2.1. Inclusion Criteria

Patients included in this study had an age over 60 years old, recent hip fracture, and a signed consent form to enroll in the study (Figure 1).

#### 2.2.2. Exclusion Criteria

Excluded patients were those under 60 years of age, those with severe cognitive impairment unaccompanied by a family member or caregiver, or those who did not provide informed consent to participate in the study (Figure 1).

### 2.3. Data Collection and Measures

#### 2.3.1. Procedures

The geriatric assessment was performed by the medical staff involved in the study, at admission, and at 72 h postoperatively. It is worth mentioning that a single evaluator conducted all assessments in order to minimize discrepancies between test administrators.

The objective of the study was to quantify the degree of frailty, pre- and postoperatively, in senior patients with hip fracture, as well as to determine if geriatric evaluation can predict postoperative functional recovery and provide insight into postoperative survival.

In this intermediate analysis, we assessed the correlations between one-year postoperative mortality and scores obtained from the preoperative geriatric evaluation (MMSE, GDS, and MNA), as well as other variables such as age, gender, and marital status.

#### 2.3.2. Geriatric Tools

All tests used in the assessment of patients are widely used instruments: for the assessment of frailty: the Groningen Frailty Indicator (GFI); for the assessment of cognitive status: the Mini Mental State Examination (MMSE); for the assessment of depression: the Geriatric Depression Scale (GDS); and for the assessment of nutritional status: the Mini Nutritional Assessment (MNA).

We established thresholds for cognitive disorders at MMSE < 25; impaired psycho-emotional satisfaction (depression) at GDS > 5; impaired nutritional status at MNA < 23.5; and frailty at GFI > 4.

Cognitive status was assessed using the Mini-Mental State Examination (MMSE), a widely used 30-point questionnaire evaluating orientation, memory, attention, language, and visuospatial skills, developed by Folstein et al. [40] The MMSE is commercially available through PAR Inc., Lutz, FL, USA. The MMSE ranges from 0 to 30, with scores ≥ 25 indicating normal cognition, 20–24 mild impairment, 10–19 moderate impairment, and <10 severe impairment. A score below 25 points was considered indicative of cognitive impairment, consistent with prior studies in elderly populations [40].

Depressive symptoms were screened using the Geriatric Depression Scale (GDS), a validated tool specifically developed for older adults and distributed by Stanford University, Stanford, CA, USA. The GDS ranges from 0 to 15, with scores of 0–4 indicating normal mood, 5–8 mild depression, 9–11 moderate depression, and 12–15 severe depression. A score greater than 5 points on the 15-item version was used to identify patients with possible depression, according to standard recommendations [41].

Nutritional status was evaluated with the Mini Nutritional Assessment (MNA), a comprehensive instrument assessing dietary intake, weight loss, mobility, psychological stress, and BMI, developed by the Nestlé Nutrition, Vevey, Switzerland. The MNA ranges from 0 to 30, with scores ≥ 24 indicating normal nutrition, 17–23.5 risk of malnutrition, and <17 malnutrition. A score below 23.5 indicates risk of malnutrition, as defined in the original validation studies [42].

Frailty was assessed using the Groningen Frailty Indicator (GFI), a multidimensional questionnaire encompassing physical, cognitive, social, and psychological domains), developed at the University of Groningen, Groningen, The Netherlands. A GFI score greater than 4 was used as the cut-off for frailty, in line with its original validation [43].

All instruments used in this study are internationally recognized screening tools for geriatric assessment.

### 2.4. Data Analysis

Data were collected by a team of physicians and analyzed using IBM SPSS Statistics software, version 18.0 (IBM Corp., Armonk, NY, USA) software. Internal consistency was calculated for all items of each rating scale.

The homogeneity of the series of values, both preoperative and postoperative, of MMSE, GDS and GFI scores allowed us to apply significance tests for continuous variables. The quantitative or qualitative interpretation of the data employed multivariate analysis, multiple linear regression, receiver-operating characteristic (ROC) curve, Spearman Skewness Test, Paired-Samples T test, and the Wilcoxon Signed-Rank Test.

We used multiple regression as a method of predicting the values of a dependent variable from the values of several independent variables. The model is built up, with the criterion for introducing a new variable being the LR (Likelihood Ratio) test. Statistical significance was defined at the 95% confidence interval (*p* < 0.05).

A logistic regression model was used to analyze the association between the independent variables and postoperative mortality. The model was built stepwise (forward selection), with the inclusion of variables based on the Likelihood Ratio (LR) test criteria for improved model fit. The independent variables considered for inclusion were the MMSE (Mini-Mental State Examination), GDS (Geriatric Depression Scale), IGF (Instrumental Geriatric Function), MNA (Mini Nutritional Assessment), the number of medications, and the number of comorbidities.

## 3. Results

### 3.1. General Characteristics

The study group’s age range was from 61 to 96 years, with an average value of 77.62 (±8.65) years, close to the median value (80 years). This suggests that the series of age values was homogeneous, favoring the application of statistical significance tests.

Of the study group, 29 (48.3%) had ages that exceeded the median value, 39 (65.0%) were female, and 38 (63.3%) were not married (Table 1).

### 3.2. Postoperative Evolution

#### 3.2.1. Cognitive Status

The mean Mini Mental State Examination (MMSE) scores declined from 22.18 preoperatively to 21.53 postoperatively (*p* = 0.126). The MMSE scale ranges from 0 to 30, with scores of 25–30 considered normal cognitive function, 20–24 indicating mild cognitive impairment, 10–19 moderate impairment, and below 10 severe impairment. Mild–moderate cognitive impairment was the most common finding both preoperatively (61.7%) and postoperatively (68.3%) (*p* = 0.889) (Table 2).

#### 3.2.2. Depression

Patients recorded slightly higher mean Geriatric Depression Scale (GDS) scores postoperatively (6.77 vs. 6.63; *p* = 0.214), where higher scores indicate more severe depressive symptoms. The GDS scale ranges from 0 to 15, with scores of 0–4 considered normal mood, 5–8 indicating mild depressive symptoms, 9–11 moderate depression, and 12–15 severe depression. The proportion of patients with mild depression increased slightly postoperatively (55% vs. 55.8%; *p* = 0.918) (Table 2).

#### 3.2.3. Nutritional Status

Nutritional status remained unchanged postoperatively. Malnutrition was found in 6.7% of patients, while 56.7% were at risk, and 36.7% had normal nutritional status both pre- and postoperatively. The MNA scale ranges from 0 to 30, with scores of 24–30 indicating normal nutritional status, 17–23.5 suggesting a risk of malnutrition, and below 17 indicating malnutrition. No statistically significant difference was observed (*p* = 1.000).

#### 3.2.4. Frailty

The postoperative mean score for frailty was markedly higher than the preoperative score (7.33 vs. 5.18), and statistically significant (*p* = 0.001). Of preoperative patients, 65% were frail, increasing postoperatively to 86.7% (*p* = 0.005) (Table 2).

Within the study group, age > 80 years and unmarried status were associated with frailty (*p* = 0.04; *p* = 0.03).

Preoperative assessments for cognitive status (*p* = 0.01), psycho-emotional status (*p* = 0.01), and nutritional status (*p* = 0.03) were significantly correlated with frailty. Also, mild–moderate cognitive impairment (*p* = 0.017), mild–moderate depression (*p* = 0.01) and malnutrition (*p* = 0.04) proved to be important predictors of frailty.

Postoperative assessment showed only cognitive status (*p* = 0.01) and psycho-emotional status (*p* = 0.01) to be significantly correlated with frailty. Also, only mild–moderate cognitive impairment (*p* = 0.04) and mild–moderate depression (*p* = 0.01) were important predictors of postoperative frailty.

Preoperative GFI was associated with preoperative MMSE (*p* = 0.01; r = −0.481), GDS (*p* = 0.01; r = 0.503) and MNA (*p* = 0.01; r = −0.382) scores (Table 3).

Postoperative GFI was significantly associated with MMSE (*p* = 0.01; r = −0.402) and GDS (*p* = 0.006; r = 0.352) scores, while its association with the number of medications did not reach statistical significance (*p* = 0.08; r = 0.339) (Table 3). The calculations used Pearson′s correlation coefficient.

#### 3.2.5. Correlation and Predictive Analysis

In the multivariate analysis of predictors of postoperative frailty, preoperative GDS scores were repeatedly found to be statistically significant in different comparisons (*p* < 0.05) (Table 4).

**Figure 2:**
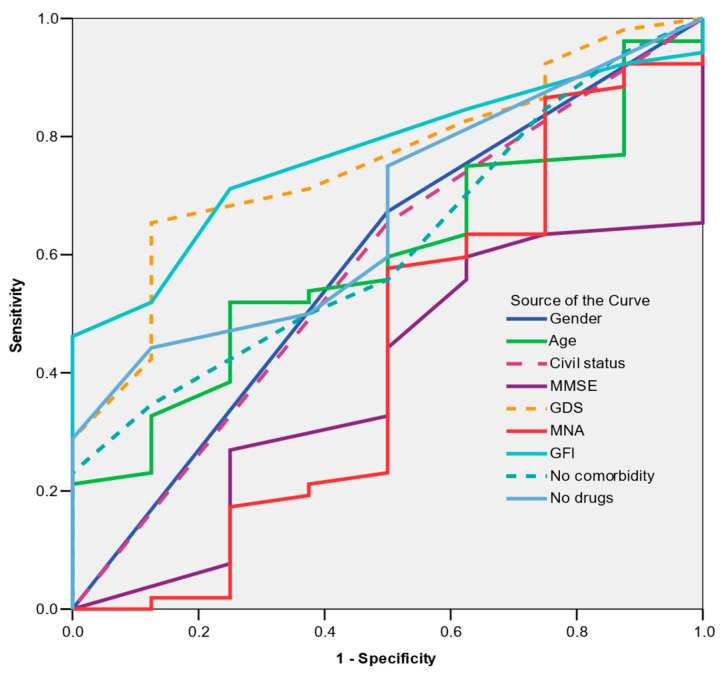
ROC curve. Predictors of postoperative frailty–preoperative frailty. Area > 0.9—excellent; 0.9 > area > 0.8—very good; 0.8 > area > 0.7—good; 0.7 > area > 0.6—fair; area < 0.6—the model is rejected.

Among the variables examined, preoperative frailty showed the highest predictive value for postoperative frailty, albeit with moderate discriminative ability (AUC = 0.768; CI = 95%: 0.635–0.901; *p* = 0.015) (Figure 2).

#### 3.2.6. Mortality Outcomes

The overall mortality rate of the study group is 42.6%. Approximately one-half died in the first year after surgery, with the highest mortality rate (42%) in the first 3 months and the lowest (5%) between 4 and 6 months after the intervention. The majority of postoperative mortality was recorded 1 year after the intervention (53%) (Figure S1, Supplementary Materials). We used intervals specific to mortality rate timeframes.

The percentage distribution of mortality did not vary significantly by sex or marital status, but it did reflect an estimated fourfold greater risk in the age group over 80 years (RR = 3.98; 95% CI: 1.96–8.06; *p* = 0.001) (Table 5).

Preoperative scores for the MMSE (*p* = 0.001, value = 3.78), MNA (*p* = 0.021, value = 7.72), and GDS (*p* = 0.046, value = 3.99) were statistically significantly correlated with postoperative mortality. Only the postoperative MMSE score was statistically correlated with mortality (*p* = 0.026, value = 11.08). The number of medications (*p* = 0.128, F = 2.36), the number of comorbidities (*p* = 0.129, F = 2.35) and postoperative psycho-emotional status (*p* = 0.601, value = 1.87) did not influence mortality (Table S1, Supplementary Materials).

The Chi square test Likelihood Ratio reported in Table S1 assesses associations between two sets of qualitative (categorial) variables, not quantitative measures; it is a hypothesis test that involves comparing the goodness of fit of two competing statistical models, typically one found by maximization over the entire parameter space and another found after imposing some constraint, based on the ratio of their likelihoods.

The current focus, four years after the last mortality assessment, is on continued monitoring of mortality rates, as well as institutionalization versus recovery of autonomy among surviving patients.

Among patients with postoperative frailty (*n* = 51), 23 died. In patients with frailty and a moderate-to-severe preoperative MMSE score, the risk of death was 4.77 times higher (OR = 4.77; 95% CI: 1.40–10.9; *p* = 0.011).

In patients with frailty and a preoperative MNA score indicating malnutrition, the risk of death was 1.48 times higher, but this was not statistically significant (OR = 1.48; 95% CI: 0.46–4.76; *p* = 0.515).

Similarly, in patients with frailty and a preoperative GDS score indicating depression, the risk of death was 1.13 times higher but not statistically significant (OR = 1.13; 95% CI: 0.33–3.92; *p* = 0.844) (Table S2, Supplementary Materials).

## 4. Discussion

A key finding of our study is the observed increase in frailty shortly after hip fracture surgery in elderly patients. This highlights the vulnerability of this population and the dynamic nature of frailty, which can worsen acutely in the postoperative period. Continuous monitoring through comprehensive geriatric assessment is essential for timely identification of these changes. According to recent data, rapid postoperative evaluation is an accurate and necessary complement to the preoperative assessment, ensuring benefits for the optimal recovery of the elderly patient with a hip fracture [44].

### 4.1. Frailty as a Prognostic Marker

Our results emphasize that preoperatively, 65% of the patients were frail, and the proportion increased postoperatively to 86.7%. These findings align with previous studies, which report frailty rates ranging from approximately 50% to over 80% in older patients undergoing orthopedic surgery, including hip fracture repair and joint arthroplasty [9,10,45]. As observed in our cohort, patients with preoperative GFI scores > 4 tended to maintain elevated frailty scores after surgery, reflecting persistent vulnerability despite surgical intervention. Prior research has also demonstrated that frailty is a stronger predictor of postoperative complications, prolonged hospital stay, and unfavorable discharge destinations than chronological age alone or other commonly cited factors [9,41,46]. In other words, frailty matters, and its identification in the preoperative stage can improve the individualization of treatment, through risk stratification, early medical decision making, prognostic counseling, and long-term care planning [9,10,45,46].

At the same time, our results have shown that the age segment over 80 years and unmarried marital status were associated with frailty. This association may reflect the cumulative biological vulnerability in individuals over 80 years of age and the potential impact of reduced social support in unmarried individuals, which can weaken their resilience and limit their ability to cope with stressors such as acute illness or surgery. Social isolation and lack of caregiving resources contribute significantly to the increased risk of frailty in older adults [4,14].

Most published studies demonstrate that frailty in older patients with hip fracture is associated with factors including advanced age, social condition, prolonged hospitalizations, comorbidities (particularly respiratory comorbidities associated with opioid analgesics), and surgical treatment [3,4,14,20]. As for the intervention itself or the surgical method, there are both findings and reservations regarding an association with frailty [9,10,46]. Nonetheless, numerous studies support surgery as the approach of choice in the older patient with hip fracture [20,30,31,32,33].

We identified an association of frailty with a series of amendable conditions such as cognitive, psycho-emotional and nutritional status, poly-medication, and the number of comorbidities. This finding emphasizes the need for a targeted therapeutic approach that might prevent frailty or reduce its degree by combating modifiable factors, such as, among others, malnutrition. Specifically, malnutrition can be addressed through individualized nutritional assessments, tailored dietary interventions, oral supplementation, and, when necessary, enteral nutrition support. Cognitive impairments may benefit from early cognitive rehabilitation programs and ongoing neuropsychological support. Psycho-emotional factors, such as depression and anxiety, should be managed with appropriate psychological counseling and, if needed, pharmacotherapy. Polypharmacy requires regular medication reviews to minimize potentially inappropriate prescriptions and reduce adverse drug reactions. Additionally, optimizing management of comorbidities through coordinated care can further mitigate frailty severity.

Our findings are consistent with published studies that identify impaired nutritional status as a significant predictor of frailty in older adults with hip fractures. The MNA is recognized as the most reliable test in predicting both prefrailty and frailty [27]. Preoperative malnutrition has been shown to independently increase the risk of postoperative mortality in this population [16], while nutritional risk indices correlate strongly with in-hospital mortality [29]. Additionally, serum albumin levels and comprehensive nutritional evaluation are important prognostic factors for postoperative complications and long-term mortality [47,48]. The combined presence of malnutrition and frailty further amplifies the risk of adverse outcomes after hip surgery [49]. These findings emphasize the critical need for early nutritional assessment and targeted interventions as part of a multidisciplinary approach to improve recovery and prognosis in frail elderly patients with hip fractures.

Nutritional interventions, including protein supplementation and vitamin D administration, are often used to combat sarcopenia (loss of muscle mass) and support overall recovery. Thus, in older adults with hip fractures, nursing care plans may need to be adjusted in accordance with scores from geriatric assessment. This may include initiating collaboration with physiotherapists to begin physical or nutritional therapy, monitoring oral calcium and vitamin D, managing the risk of depression through early identification of mood disturbances, maintaining empathetic communication with both the patient and their family, and promoting a realistic yet optimistic outlook on recovery and regaining autonomy. Proper nutrition is essential for muscle repair and the reduction of frailty. It is also important to optimize medication management to minimize polypharmacy and adverse drug interactions [12,14].

Moreover, we identify that the nutritional status did not change postoperatively in the study group. We consider this aspect as a consequence of the low sensitivity of the short-term MNA test. Thus, a 72 h postoperative MNA test cannot accurately assess the impact of surgery on nutritional status. Other, more targeted data report the benefit of combating malnutrition and frailty in post-interventional recovery [49,50,51]. A previous cohort study conducted on a group of patients who needed hip arthroplasty showed that both frailty and malnutrition cumulatively and negatively influence the recovery process [49]. Other data are much more specific in recognizing that malnutrition in frail patients with hip fracture correlates with negative postoperative results, constituting a modifiable risk factor, compared to frailty [51].

Independent of the pathophysiological or clinical approach to frailty syndrome, applying this concept to the older population with hip fracture reflects a host of other problems with an impact on therapeutic decision and recovery. Examples are the socio-economic condition of the older patient, who usually face limited material resources and might lack family support [6]. According to statistics, between 2000 and 2050, the proportion of the population over 60 is expected to double from 11% to 22%, and the number of older adults who cannot care for themselves will increase fourfold in developing countries [8,52].

### 4.2. Role of Cognitive and Emotional Status

Cognitive impairment, reflected by MMSE scores < 25, and psycho-emotional depression, reflected by GDS scores > 5, were found in our results to be important predictive factors for frailty. According to our data, only mild–moderate cognitive impairment and mild–moderate depression proved to be important predictors of postoperative frailty.

In elderly patients with neurocognitive impairment, detecting pain requires a careful history and detailed assessment. The geriatric nurse is often the first to identify physiological and behavioral changes induced by pain—such as grimacing, confusion, antalgic posturing, and vasovagal responses—thereby performing a key role in timely intervention.

Regarding cognitive impairment, some previous studies have shown that it was a predictor of poor functional recovery after hip fracture [53,54,55,56,57]. Some recent data demonstrate the important role of cognitive function in postoperative frailty in older persons who have suffered a hip fracture [6]. Also, a meta-analysis based on eight cohort studies, which included 222 patients, aimed to identify factors associated with mortality in older patients with hip fracture and showed that cognitive impairment, including delirium, was significantly correlated with a negative prognosis in these patients [54].

A significant proportion of patients required admission to the Intensive Care Unit (ICU), which can be attributed to the combined impact of multiple perioperative stressors. Beyond the physiological burden of the surgical procedure itself, factors such as acute postoperative pain, the use of sedative analgesics (which may contribute to respiratory depression and delirium), and the abrupt reduction in functional independence likely intensified underlying frailty in these patients. This multifactorial vulnerability, particularly common among older adults with pre-existing deficits, highlights the need for close monitoring and specialized postoperative care, often necessitating ICU-level support to ensure hemodynamic stability, prevent complications, and facilitate early recovery.

Postoperative mortality is a key indicator in evaluating the prognosis of geriatric patients with hip fractures. The integration of a complex geriatric assessment in the preoperative period can contribute to the early identification of death risk and the timely adjustment of the therapeutic plan [54].

The obtained data confirm the recent literature showing that cognitive disorders, depression, and malnutrition are significant predictors of postoperative mortality in geriatric patients with hip fractures. These results support the importance of complex geriatric assessment in the preoperative period, thereby improving risk stratification [54].

Even though our results showed that mild–moderate impaired cognitive status is associated with postoperative frailty, according to other results in the literature, delirium is significantly associated with poor postoperative recovery after hip fracture, regardless of the interventions performed on preoperative frailty [57].

As for depression, several studies confirm our findings, suggesting that depression in the older patient with hip fracture is associated with frailty [55,56,57]. The results of a study show that depression increases mortality and the degree of frailty in older individuals, independent of the existence of a traumatic physical event such as a hip fracture [55]. However, although depression is correlated with poor outcomes during recovery after hip fracture surgery, some data suggest that preoperative depression identification methods could be improved, and the prognostic capabilities of depression in older adults are still being researched [56].

### 4.3. Polypharmacy Relevance

We identify that the number of medications was statistically significant, in comparison with all the other predictive parameters of postoperative frailty. Several articles report that anticoagulants have prognostic value with postoperative frailty [15,58,59,60]. DOAC anticoagulants are most frequently found in the medication of the older patient. The literature shows that a multidisciplinary team can play a crucial role in addressing the risks and benefits of perioperative DOAC/anti-vitamin K anticoagulation in older adults with hip fracture [15]. Another study draws attention to issues with timing of surgical interventions in older patients taking anticoagulants [58].

A study demonstrated that beta-blockers are associated with an improvement in prognosis at 30 days postoperative in frail older patients with hip fracture. Although our results were only related to the number of drugs and not to their classes, we acknowledge the frequency of beta-blockers in the treatment of older individuals with chronic conditions [59].

However, a number of studies identified positive associations between frailty and polypharmacy, predicting institutionalization and negative outcomes in older adults [11,12,19,20,61,62]. These results suggest the merit of deprescribing in the frail patient, independent of the existence of a traumatic event, as a simple intervention that can lessen frailty [61].

### 4.4. Clinical Implications for Multidisciplinary Care

Recent approaches to postoperative management for senior hip fracture patients emphasize the importance of early mobilization and individualized rehabilitation programs in mitigating frailty progression and improving overall outcomes. A holistic strategy that integrates multidisciplinary care—including comprehensive geriatric assessments, nutritional support, and community-based rehabilitation—addresses not only physical recovery but also cognitive and psychosocial challenges. This comprehensive care model optimizes resource allocation while ensuring that interventions are tailored to the specific needs of each patient [12,14,20].

Empirical evidence from recent studies suggests that rapid postoperative evaluation and early mobilization are associated with significant improvements in functional recovery and reductions in postoperative complications. The integration of structured, multidisciplinary rehabilitation strategies appears to enhance patient outcomes and quality of life, underscoring the clinical relevance of a coordinated, patient-centered approach in the management of senior patients following hip fracture surgery.

Focused exercises to rebuild muscle strength and improve balance, techniques to reduce fall risk and enhance mobility, and activities that improve cardiovascular health and overall stamina and strength are important rehabilitation strategies [20].

International guidelines emphasize the importance of structured perioperative care for older surgical patients. The American Geriatrics Society (AGS), in collaboration with the American College of Surgeons, advocates for a standardized preoperative approach based on the Comprehensive Geriatric Assessment (CGA), targeting key domains such as cognitive and functional status, nutritional risk, polypharmacy, and delirium prevention [63]. Similarly, the European Geriatric Medicine Society (EUGMS), through its Special Interest Group in Perioperative Medicine, promotes coordinated orthogeriatric co-management, research collaboration, and the development of consensus-based clinical pathways. Both initiatives underline the need for multidisciplinary strategies to optimize surgical outcomes in older adults [64].

## 5. Conclusions

The institution of early surgical treatment in the frail older patient with hip fracture remains controversial due to the lack of prospective data on large patient samples. Our study adds valuable prospective evidence on this vulnerable population, highlighting the importance of complex geriatric assessment to better understand postoperative outcomes.

Our findings highlight the significant impact of frailty on postoperative recovery and complications in older adults with hip fractures, underscoring the need for tailored multidisciplinary care. Geriatricians, orthopedic surgeons, nurses, physical therapists, nutritionists, and occupational therapists must collaborate closely to optimize preoperative evaluation, manage comorbidities, ensure early mobilization, and provide personalized rehabilitation. By integrating these specialties, patient outcomes are significantly improved through better coordination of care, tailored interventions, and proactive management of frailty.

A multidisciplinary geriatric evaluation aids early identification of high-risk patients, improving prognostic accuracy. Integrating geriatric assessment in the preoperative stage not only allows for estimating the potential for functional recovery but also for predicting postoperative mortality. This approach underscores the need for standardizing geriatric evaluation in the care of elderly patients with hip fractures.

In conclusion, functional assessment and frailty evaluation are essential in determining the most suitable recovery path for senior patients. The presence of comorbidities, cognitive disorders, depression, and malnutrition plays a significant role in shaping the potential for recovery.

## 6. Limitation of the Study

We acknowledge the small sample size as a limitation of the study, attributed to the COVID-19 pandemic. While timely and clinically relevant, the study remains preliminary due to its small sample and interim nature. A larger cohort with longer follow-up is essential in order to confirm impact. Consequently, the generalizability of the results is limited, and further studies with larger cohorts are warranted.

Another limitation of the study through the impact of the COVID-19 pandemic was the restricted possibility of conducting postoperative follow-up evaluations beyond the initial hospitalization period. Moreover, the clinical condition of postoperative patients during the first 72 h—marked by cognitive and organic deterioration, the effects of surgical stress, acute pain, sedative medication, and temporary loss of autonomy—limited the feasibility of accurate assessment of early-phase postoperative frailty. These factors justified the need for continuous monitoring in the Intensive Care Unit instead of conducting functional assessments that could have yielded unreliable results.

Nevertheless, we recognize that the short term of the follow-up limits our understanding of medium- and long-term clinical outcomes. This study is ongoing, and further follow-up of the same patient cohort is planned to assess their functional recovery, reintegration into their socio-familial environment, and long-term survival. Future results will be analyzed and published to provide a more comprehensive picture of the recovery trajectory of these patients.

The exclusion of 34 patients due to early postoperative deterioration and ICU transfer may have introduced selection bias, more specifically, survivorship bias. This could lead to an underestimation of postoperative complication severity and functional decline, as the frailest individuals—likely to have worse outcomes—were not included in the analysis. Consequently, the generalizability of our results is limited to patients who had a relatively more favorable early postoperative course. Future prospective studies should aim to include all postoperative patients, using early proxy assessments or adjusted analytic strategies. This would improve the capture of outcomes in this high-risk subgroup.

The assessment timing (72 h post-surgery) may not capture the full impact of surgical and pharmacologic interventions on nutritional or cognitive parameters. In the immediate postoperative period, acute physiological stress, pain, sedation, and temporary delirium or functional decline are common in elderly patients, potentially masking meaningful changes in baseline frailty, nutrition, or cognition. Consequently, evaluations at this stage might reflect transient postoperative effects rather than true recovery or decline. This limits the sensitivity and clinical interpretability of some geriatric assessment tools when applied so early after surgery. Future phases of the study will include longitudinal assessments at 30 and 90 days, allowing for a more accurate evaluation of medium-term recovery trajectories and the potential reversibility or persistence of postoperative deficits.

Another important limitation of the present study is the absence of a comparative or control group. Without a matched control cohort or comparison with standard care, the interpretative power of our findings regarding the specific benefits of comprehensive geriatric assessments remains limited. As an observational and exploratory study, our primary objective was to assess the feasibility and predictive value of geriatric assessments in the early postoperative period. However, we acknowledge that comparative analyses with matched control groups would strengthen the causal inferences. They would clarify whether the observed outcomes are attributable to the geriatric assessment approach itself, or to other clinical factors. Future studies are warranted to include comparative analyses with standard care, or alternative assessment methods, to better delineate the clinical impact of comprehensive geriatric evaluation on postoperative recovery and long-term outcomes.

This study represents an initial analysis of prospectively collected data from an ongoing observational cohort. While prospective design minimizes recall bias, selection bias may still arise due to incomplete follow-up data for some patients. We addressed this by including only participants with complete data on the variables of interest. These limitations are acknowledged when interpreting the findings.

## Figures and Tables

**Table 1:** General characteristics of the study group.

Characteristics	Study Group *n* = 60	
	*n*	%
**Female**	39	65.0
**Male**	21	35.0
**Age**		
**Mean ± SD**	77.62 ± 8.65	
**Median/min–max**	(79/61–96)	
**<80 years**	31	51.7
**≥80 years**	29	48.3
**Unmarried**	38	63.3
**Married**	22	36.7
**Polypharmacy (#)** **Median/min–max**	3/0–8	
**Comorbidities (#)** **Median/min–max**	4/1–11	

Abbreviations: SD, standard deviation; #, number.

**Table 2:** Postoperative evolution of cognitive impairment, emotional status, nutritional status, and frailty.

Score Evaluation	Preoperative	Postoperative	Significance Test	*p*
**MMSE** **median ± SD** **median/min–max**	22.18 ± 5.69 23/5–30	21.53 ± 5.52 22/9–30	Paired-Samples T test Mood′s Median Test	0.126 0.003
**Cognitive impairment** **Severe** **Moderate** **Mild** **Without impairment**	2 (3.3%) 16 (26.7%) 21 (35.0%) 21 (35.0%)	2 (3.3%) 18 (30.0%) 23 (38.3%) 17 (28.3%)	Chi Square Test Likelihood Ratio	0.889
**GDS** **mean ± SD** **Median/min–max**	6.63 ± 3.49 6.50/0–14	6.77 ± 3.33 7/0–14	Paired-Samples T test Mood′s Median Test	0.214 0.002
**Psycho-emotional impairment (depression)** **Severe** **Mild** **Normal**	7 (11.7%) 33 (55.0%) 20 (33.3%)	8 (13.3%) 34 (55.8%) 18 (30.0%)	Chi Square Test Likelihood Ratio	0.918
**MNA** **mean ± SD** **Median/min–max**	22.61 ± 3.28 23/14.2–29.0	22.61 ± 3.28 23/14.2–29.0	Paired-Samples T test Mood′s Median Test	1.000 -
**Nutritional status** **Malnutrition** **At risk of malnutrition** **Normal**	4 (6.7%) 34 (56.7%) 22 (36.7%)	4 (6.7%) 34 (56.7%) 22 (36.7%)	Chi Square Test Likelihood Ratio	1.000
**GFI** **mean ± SD** **Median/min–max**	5.18 ± 3.09 6/0–13	7.33 ± 3.20 7/1–13	Paired-Samples T test Mood′s Median Test	**0.001** 0.106
**Frailty** **Frail** **Robust**	39 (65.0%) 21 (35.0%)	52 (86.7%) 8 (13.3%)	Chi Square Test Likelihood Ratio	**0.005**

Abbreviations: SD, standard deviation; min/max, minimum and maximum.

**Table 3:** Correlation between frailty and analyzed parameters.

GFI	Preoperative		Postoperative	
	r	*p*	r	*p*
**MMSE**	−0.481	<0.01	−0.402	0.001
**GDS**	0.503	<0.01	0.352	0.006
**MNA**	−0.382	<0.01	−0.253	0.051
**No Comorbidities**	0.058	0.658	0.089	0.500
**No Medications**	0.224	0.085	0.339	0.008

**Table 4:** Multivariate predictors of postoperative frailty.

Model	Unstandardized Coefficients		Unstandardized Coefficients	T	*p*	95% Confidence Interval for β
	β	Er. Std.	Beta			
**Constant**	3.830	7.387		0.518	0.606	−9.625 ÷ 20.944
**Sex**	0.147	0.892	0.022	0.165	0.870	−1.545 ÷ 2.066
**Age**	0.001	0.057	0.004	0.024	0.981	−0.119 ÷ 0.112
**Marital Status**	0.997	0.877	0.152	1.137	0.261	−0.780 ÷ 2.742
**MMSE pre**	−0.070	0.093	−0.125	−0.758	0.452	−0.256 ÷ 0.116
**GDS pre**	0.288	0.136	0.313	2.109	**0.040**	−0.011 ÷ 0.543
**MNA pre**	0.020	0.140	0.021	0.144	0.886	−0.344 ÷ 0.267
**# comorbidities**	−0.074	0.168	−0.060	−0.443	0.660	−0.416 ÷ 0.258
**# medications**	0.357	0.168	0.290	1.930	0.083	−0.004 ÷ 0.675

Abbreviations: pre, preoperative; #, number of.

**Table 5:** Analysis of mortality by demographic characteristics.

Characteristics	Death *n* = 40 (42.6%)		Survival *n* = 54 (57.4%)		Chi2 Test	*p*	Estimated Risk	CI95%
	*n*	%	*n*	%				
**Female**	14	35.0	16	29.6	0.304	0.581	1.15	0.71–1.86
**≥80 years**	33	82.5	18	33.3	23.79	0.001	3.98	1.96–8.06
**Unmarried**	30	75.0	31	57.4	3.186	0.074	1.37	0.98–1.92

## Data Availability

The original contributions presented in this study are included within the article/Supplementary Materials. Further inquiries can be directed to the corresponding author.

## Supplementary Materials

The following supporting information can be downloaded at: https://www.mdpi.com/article/10.3390/nursrep15070262/s1, Figure S1. Postoperative mortality rate. Table S1. Correlations of MMSE, GDS, MNA scores, number of medications, number of comorbidities with mortality. Table S2. Mortality risk associated with preoperative MMSE, GDS, MNA scores in patients with postoperative frailty.

## Public Involvement Statement

There was no public involvement in any aspect of this research.

## Guidelines and Standards Statement

This study follows the STROBE guidelines for observational studies.
