# Supplementary material for: Geriatric Assessment as an Important Tool for Post-Hip Surgery Prognosis in Seniors
Source: Nurs Rep. 2025 Jul 17;15(7):262. doi: 10.3390/nursrep15070262 (PMC12299817; doi:10.3390/nursrep15070262)
Supplement: Supplementary file 1 [file nursrep-15-00262-s001.zip › nursrep-3710965-supplementary.pdf]

Supplementary Materials:

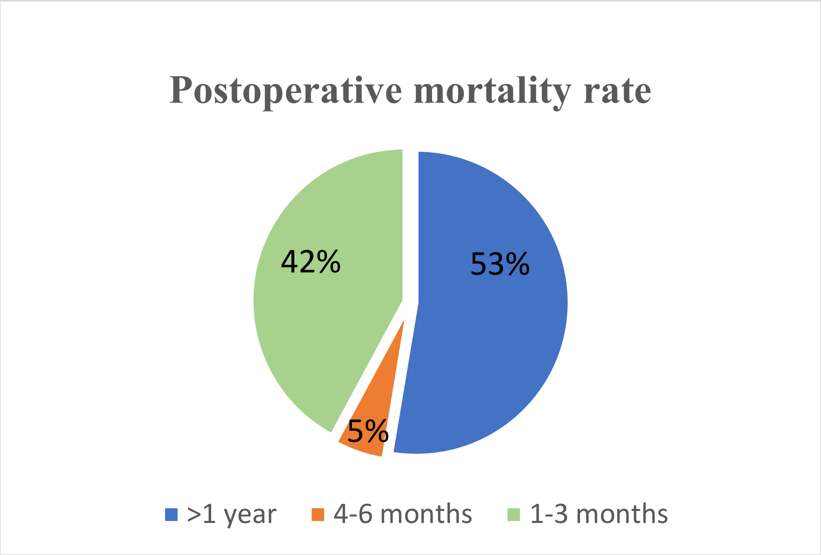

Figure S1. Postoperative mortality rate

Table S1. Correlations of MMSE, GDS, MNA scores, number of medications, number of comorbidities with mortality

Score	Mortality (n=40)		Survival (n=54)		Chi square test Likelihood Ratio
	n	%	n	%	
**pre-operative**					
MMSE					value=3,78
severe	8	20,0	1	1,9	p=0,001
moderate	17	42,5	9	16,7	
mild	9	22,5	19	35,2	
normal	6	15,0	25	46,3	
GDS					value=3,99
normal	8	20,0	17	31,5	p=0,046
mild	20	50,0	30	55,6	
depressed	12	30,0	7	13,0	
very depressed					
MNA					value=7,72
Risk of malnutrition	10	25,0	3	5,6	p=0,021
	19	47,5	29	53,7	

malnutrition<br>normal	11	27,5	22	40,7	
**post-operative**					
MMSE<br>severe<br>moderate<br>mild<br>normal	 2<br>11<br>7<br>3	 5,0<br>27,5<br>17,5<br>7,5	 1<br>6<br>16<br>14	 1,9<br>11,1<br>29,6<br>25,9	value=11,08<br>p=0,026
GDS<br>normal<br>mildly depressed<br>very depressed	 7<br>12<br>5	 17,5<br>30,0<br>12,5	 12<br>21<br>4	 22,2<br>38,9<br>7,4	value=1,87<br>p=0,601

Score	Mortality(n=40)		Survival (n=54)		FANOVA test
	n	%	n	%	p
Nr.of drugs ±SD<br>limits	4 ± 2<br>0 - 7		3 ± 2<br>0 - 8		F=2,36<br>p=0,128
Nr.of comorbidities ±SD<br>limits	6 ± 3<br>2-13		5 ± 2<br>1-10		F=2,35<br>p=0,129

Table S2. Mortality risk associated with preoperative MMSE, GDS, MNA scores in patients with postoperative frailty

Score		Postoperative frailty				Chi2 test p	OR	CI 95%
		Death (n=23)		Survival (n=28)				
		n	%	n	%			
Preoperative MMSE	moderate-severe	13	56.5	6	21.4	0.011	4.77	1.40-10.9
Preoperative MNA	malnutrition	16	69.6	17	60.7	0.515	1.48	0.46-4.76
Preoperative GDS	depression	17	73.9	20	71.4	0.844	1.13	0.33-3.92
